# Cervical Manipulation and Koren Specific Technique Emotions Protocol in the Improvement of Intensive Nocturnal Dry Cough: A Case Report

**DOI:** 10.7759/cureus.51502

**Published:** 2024-01-02

**Authors:** Hsuan Pin Chang

**Affiliations:** 1 Chiropractic, Neuro-Spinal Center, Taichung, TWN; 2 College of Oral Medicine, Chung Shan Medical University, Taichung, TWN

**Keywords:** upper cervical spine, nocturnal dry cough, kst emotions protocol, koren specific technique, vagus nerve, neurogenic cough, spinal manipulation, depression, chronic cough, chiropractic

## Abstract

This report describes a 42-year-old female patient who presented with an intensive nocturnal dry cough persisting for over six months. Subsequent to the prolonged cough, she developed shoulder and neck discomfort, leading her to seek chiropractic care. The patient received cervical chiropractic adjustments combined with the Koren Specific Technique (KST) emotions protocol. The patient was mainly treated for her musculoskeletal complaint. However, after two treatment sessions, the patient's chronic cough showed significant improvement. Two weeks later, the cough had completely ceased, and her shoulder and neck discomfort had also improved. The cough symptoms did not reappear during the six-month follow-up. The mechanism of cough improvement remains unclear, whether it is due to spinal adjustments, the KST emotions protocol, their combined effects, or merely a placebo response. This report discusses the potential underlying mechanisms of the case improvement, suggesting a non-pharmacological adjunctive therapeutic approach that could be investigated further in future research.

## Introduction

Coughing is one of the primary reasons why patients seek medical attention, with approximately 10% of the global population experiencing issues with chronic coughing [1]. While chiropractic care primarily addresses musculoskeletal and nervous system dysfunctions, it is important to recognize the significant pressure exerted on the shoulders, neck, chest, and intervertebral discs during the Valsalva maneuver that occurs in the moment of coughing. Treating musculoskeletal issues in patients with persistent coughs is a challenge. The current evidence and literature supporting the improvement of cough through chiropractic adjustments are limited. Only one other case report documenting the improvement of cough symptoms after chiropractic treatment was found in the literature [2]. This case may be one of the first documented instances where rapid chronic cough improvement was observed after chiropractic adjustment.

A chronic cough is characterized by a persistent cough lasting over two months. After common causes of coughing are ruled out, such as respiratory infections, chronic obstructive pulmonary disease, gastroesophageal reflux disease, postnasal drip, side effects of ACE inhibitors, and pathologic causes like tuberculosis, tumors, or heart failure, patients may receive a diagnosis of chronic neurogenic cough or somatic cough syndrome. The etiology and mechanisms of chronic cough are not yet fully understood, but they may be related to neural stimulation or emotional anxiety [3-5].

Chronic coughing has a negative impact on the quality of life and mental health of patients, especially during daily activities or sleep. Patients often report a sensation of a foreign object in the throat, leading to frequent coughing without necessarily producing significant phlegm or secretions. This type of cough not only persists but can also suddenly trigger intense and frequent bouts, causing prolonged, intense contractions or tension in the associated muscle groups. This can result in discomfort in the shoulders, neck, chest, and abdomen, and even symptoms like urinary incontinence. The Valsalva maneuver during coughing moments can also lead to a sudden increase in intervertebral disc pressure, exacerbating symptoms of spinal or nerve root compression. Frequent coughing can also disrupt sleep quality, leading to anxiety, social avoidance, and depressive psychological changes. Long-term chronic coughing significantly impacts patients socially and psychologically. Therefore, it is essential to assess a patient's emotional stress and screen for depression [4].

In the treatment of chronic cough, commonly employed methods include pharmacotherapy and behavioral therapy. Pharmacotherapy primarily aims to control the cough reflex, utilizing medications such as antidepressants, antispasmodics, and sedatives [3,6]. Behavioral therapy encompasses cognitive behavioral therapy and breathing exercises, aiding patients in altering their response to coughing and their perception of pain [3,7]. Additionally, nerve block techniques may be employed to attenuate neurogenic chronic coughs resulting from excessive nerve activation [3,8].

Statistics indicate that only 28% of chronic cough patients are satisfied with conventional antitussive treatments, and 20% of patients eventually opt to discontinue treatment due to its ineffectiveness [9]. Therefore, exploring new treatment options is crucial. In this report, we present the case of a 42-year-old female patient who has been experiencing severe nocturnal coughing for six months. Despite a series of examinations and treatments, her symptoms persisted, accompanied by neck and shoulder pain. Cervical adjustment and the Koren Specific Technique (KST) emotions protocol were intended to address her musculoskeletal complaints but inadvertently led to an improvement in her chronic nocturnal cough. Currently, there is no peer-reviewed scientific literature discussing such an approach, either individually or simultaneously for chronic cough. The mechanism behind this improvement is unclear but warrants further exploration and research.

## Case presentation

Chief complaint

The patient, a 42-year-old female, presented to the chiropractic office with a complaint of soreness in the neck and shoulders due to persistent, severe nocturnal dry coughing for the past six months. The neck and shoulder pain were rated at 4/10 on the pain scale. She seldom coughed during the day, but the coughing became notably pronounced at night during deep sleep. At times, the coughing was severe enough that she had to stop sleeping and sit up for several hours before subsiding. The patient reported that the cough typically did not occur right away when she began to sleep but started after 3 a.m. and may persist until around 6 a.m. The patient experienced extremely poor sleep quality and was unable to have uninterrupted sleep at night due to persistent and intense coughing.

Childhood medical history

At the age of 10, the patient presented with a recurrent low-grade fever and a persistent cough with phlegm. A Traditional Chinese Medicine (TCM) practitioner initially evaluated her, but she was subsequently referred to a cardiac surgeon due to suspected heart issues. She was diagnosed with a streptococcal infection, resulting in rheumatic heart disease and leading to cardiac valve replacement surgery. Following the surgery, her health condition improved significantly. During the initial postoperative period, her healthcare provider prescribed daily anticoagulants and recommended receiving a monthly dose of penicillin injections until the age of 25. However, at the age of 22, due to personal choice, she decided to discontinue anticoagulant therapy and antibiotic injections but still continued to attend regular cardiac follow-up appointments. Prior to the current onset of symptoms, her overall health was good, with no medication use and no complications related to previous cardiac surgery.

Coughing history

Since November 2022, she has been experiencing nocturnal dry coughing without signs of fever or infection. After two weeks with no improvement, she sought medical attention for the first time. While primarily opting for TCM treatment, she also sought Western medical evaluation during this period. Other potential serious cardiopulmonary lesions were ruled out. Based on her past experiences, she noticed that drugs tended to alleviate one symptom but often led to the emergence of new symptoms as side effects. Due to personal treatment preferences, she declined Western pharmaceutical intervention. Her current cough has never been managed with antitussives, mucokinetics, or mucolytics, and she has no history of taking ACE inhibitors.

Throughout the treatment period, she consulted three different TCM practitioners, primarily receiving herbal remedies, acupuncture, and Tuina massage therapy. In mid-May 2023, due to the lack of noticeable improvement, she discontinued her TCM treatment. Subsequently, she briefly attempted one session of spiritual and energy-based therapies, as well as Gua Sha (an instrument-assisted soft tissue mobilization treatment). While she experienced momentary relief physically and mentally, there was no improvement in her coughing symptoms. She started her chiropractic care for the first time in June 2023.

Social history

The patient primarily engages in sedentary office work and does not have any smoking or alcohol habits. The long-standing, severe nocturnal coughing significantly disrupts her sleep as well as that of other family members. Daytime fatigue and lethargy have been causing her additional stress. She experiences feelings of depression, although she has not undergone a professional assessment or received treatment for it.

Chiropractic examination findings

Upon examination focused on the musculoskeletal system, the following observations were made. The patient exhibits anterior head carriage and rounded shoulders. A visible surgical scar extends from the sternal notch to the xiphoid process, indicating a past surgical procedure. Hypertonicity in the upper cervical spine, with limited range of motion mainly in the extension and rotations. Localized tender points with restricted joint play were identified at levels of C0 to C3. The bilateral sternocleidomastoid, levator scapulae, pectoralis minor, and pectoralis major muscles show signs of tension and have localized tender points. There was no evidence of limb edema. Deep tendon reflexes and muscle strength testing show no abnormalities. During the inquiry of medical history, physical examinations, and throughout the brief periods (10-15 minutes) of lying on the back or prone during treatment, no coughing symptoms were observed in the patient.

KST emotions protocol

**Figure 1:**
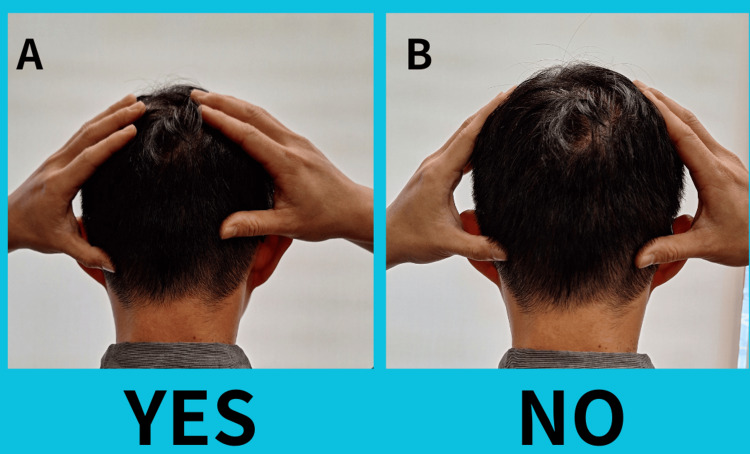
Occipital drop method (A) One occipital drop moves further down than the other: "YES" in binary biofeedback reaction. (B) Two sides stop at the same level: "NO" in binary biofeedback reaction.

The KST is a set of adjustment techniques developed by Dr. Tedd Koren in 2004. According to the KST official website, "KST is a protocol that can be applied to numerous healing arts, including chiropractic, osteopathy, medicine, dentistry, psychology, optometry, naturopathy, and others [10]." KST integrates spinal column stressology, directional non-force technique, cranio-sacral therapy, somato-emotional release, and neuro-emotional technique (NET). Similar to applied kinesiology, which uses muscle testing as a binary biofeedback device, KST employs the occipital drop (OD) method to assess patients [11]. A practitioner places hands on the back of the patient's skull and then moves them smoothly down to feel if one side appears to “drop” in relation to the other side. If one side drops lower, it indicates "Yes" in binary biofeedback. If there is no drop noted, it indicates "No" in binary biofeedback (Figure 1). Other chiropractic methods use a similar binary biofeedback approach, such as the short leg reflex in the activator method chiropractic technique [2,11]. However, it is important to note that due to the relative novelty of KST, there is currently no peer-reviewed literature available to reference its assessment methods and treatment efficacy.

The KST emotions protocol is derived from the concept of NET, based on the close connection between emotions and the body. Instead of using muscle testing in NET, KST employs the OD method to assess patients. It posits that emotions and stress can negatively impact the body, leading to pain, tension, and other issues. For example, merely thinking about a favorite food can lead to salivation, especially when memory is associated with stress. This physiological reaction is termed "conditioning," and the strength of the conditioning is determined by the intensity of the emotion. Over time, the conditioned response typically diminishes and eventually fades away through the natural process of "extinction." However, if the body is not in a balanced state during conditioning, the extinction process may be hindered. This can result in an abnormal response to similar stimuli in the future, where a once-appropriate reaction becomes unnecessary and even excessive. The KST emotions protocol or NET, primarily focuses on adjusting this mind-body balance rather than providing counseling or therapy for emotions [11].

**Figure 2:**
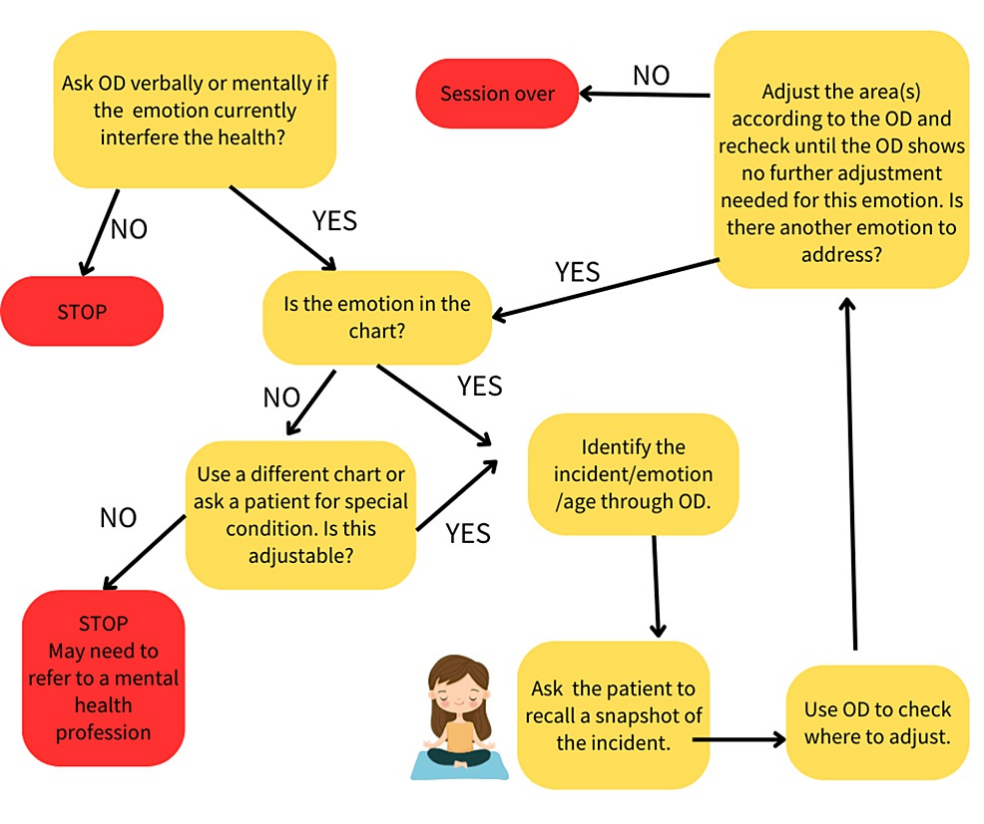
KST emotions protocol flow chart

The KST emotions protocol flow chart is shown in Figure 2. During the KST emotions protocol, the practitioner first uses the OD and KST emotions charts to identify which emotion is interfering with the body. Then, use OD to check if it is OK to address and continue to adjust the body-mind balance. If yes, the patient is asked to recall and immerse themselves in the scene, emotion, and sensation from that time in their mind. The practitioner uses OD to re-evaluate the corresponding points on the body related to that emotion and then gently touches or applies pulsing techniques to stimulate those points, resetting mind-body balance [11]. These techniques are designed to assist the body in releasing stress, promoting body-mind balance, and integration. During the process, there are instances where emotional release, such as crying, may accompany it. Typically, after the adjustment, one can experience restful sleep and a sense of relaxation both physically and mentally.

Response from KST emotions protocol

During the first two sessions of the KST emotional protocol, the patient's past emotional traumas, including significant childhood heart surgery and subsequent lengthy medical interventions, paternal infidelity, experiences of sexual harassment, and recent thoughts of self-harm due to the persistent cough, were identified through OD. Then, throughout the adjustment protocol, emotions associated with specific areas of the body, including the sternum, coccyx, clavicle, ribs, first thoracic vertebra, and temporal bone, were identified through OD as well. Corresponding light-tapping corrections were administered to these areas, eliciting strong emotional responses and tears from the patient. Following the adjustments, the patient reported an immediate sense of relaxation, both physically and emotionally. In a thank-you note she later wrote, she mentioned, "After the two sessions of the KST emotions protocol, I feel like I have regained control of my health." The patient treatment process and responses are listed in Table 1.

**Table 1:** The patient treatment process and responses C0/C1: The joint between occiput and the 1st cervical spine. C3/C4: The joint between the 3rd and 4th cervical spine. C7/T1: The joint between the 7th cervical and the 1st thoracic spine. C6/C7: The joint between the 6th and 7th cervical spine.

Visit	Day	Patient-reported status	Adjustment
1st	Day 1	The patient has been experiencing a severe nocturnal cough for over six months, leading to discomfort and soreness in the neck and shoulders. This persistent condition has also resulted in inadequate sleep and feelings of depression.	C0/C1 and KST emotions protocol
2nd	Day 3	After the first visit, the intensity and duration of the cough significantly decreased. There were two consecutive nights of good-quality sleep lasting over 6 hours.	C3/C4 and KST emotions protocol
3rd	Day 13	After the second visit, the cough has become almost negligible. There was only occasional, light coughing that didn't disrupt sleep. The discomfort in the shoulder and neck areas has also improved.	C7/T1
4th	Day 49	The patient reported that both the coughing and discomfort in the shoulders, neck, and chest have been resolved. There was occasional stiffness in the shoulders and neck. The emotional state has also improved significantly.	C0/C1 and C6/C7

**Figure 3:**
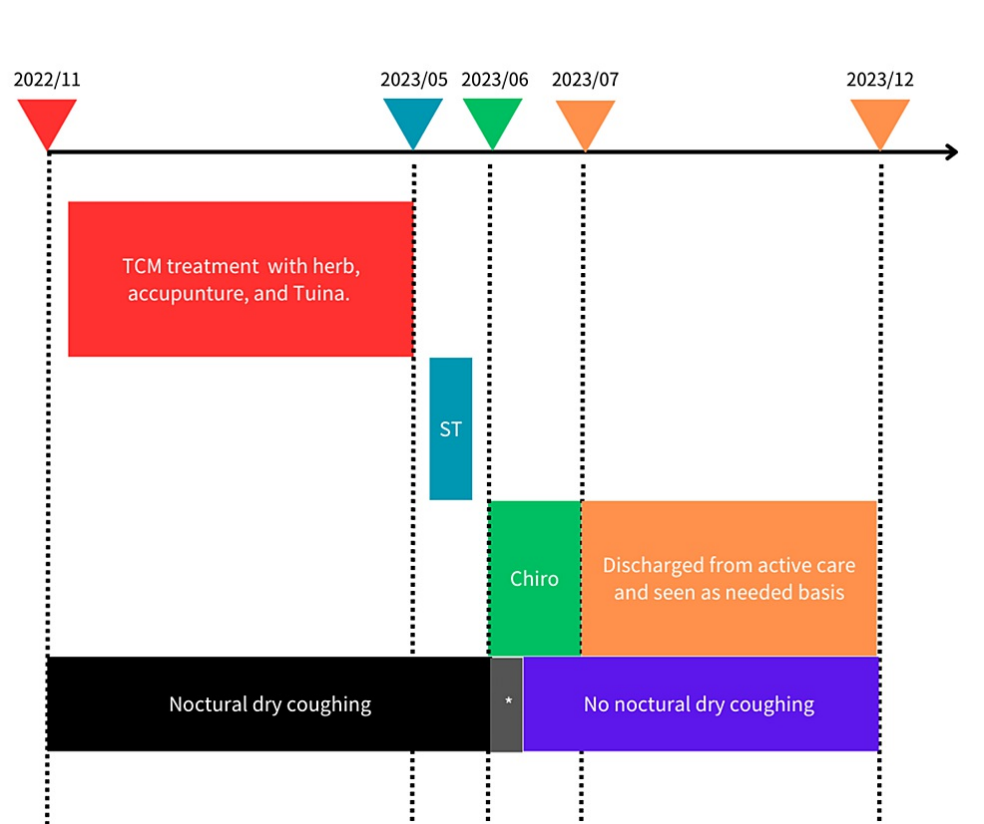
Patient timeline ST: spiritual therapies. *In gray color: Nocturnal dry coughing improving.

After the fourth visit, we discharged the patient from active chiropractic care and continued to see her on an as-needed basis. We maintained contact and followed up with her for six months. We advised her to seek further evaluation from a respiratory physician if the cough returned. During this period, she visited twice for newly onset lower back pain and wellness care. She reported no recurrence of nocturnal coughing in six months. The detailed timeline of the treatment course is shown in Figure 3.

The patient conducted a self-assessment using the Leicester Cough Questionnaire (LCQ), recognized as an effective tool for assessing the physical, psychological, and social impact of coughing. Scores range from 1 to 7, with lower scores indicating a more significant impact on quality of life. A 2022 study by Nguyen observed improvements of 0.8, 0.9, and 0.8 points in the physical, psychological, and social domains, respectively. On average, there was an increase of 1.3 to 2.3 points in the total LCQ score, signifying clinically meaningful improvements for the patients [12].

In this case, the patient's self-assessment scores prior to chiropractic treatment were 1.8 (physical), 2.3 (psychological), and 3.5 (social), resulting in an average total of 2.3. Following treatment, the self-assessment scores improved to 5.9 (physical), 6.7 (psychological), and 6.5 (social), yielding an average total of 6.3. All metrics exhibited enhancement, with a total score increase of 4 points compared to pre-treatment. This signifies a notable clinical amelioration in the patient's symptoms (Table 2).

**Table 2:** LCQ evaluation of the patient's pre- and post-treatment

LCQ	Physical	Psychological	Social	Average
Pre	1.8	2.3	3.5	2.3
Post	5.9	6.7	6.5	6.3
Difference	+4.1	+4.4	+3.0	+4.0

## Discussion

In this case, due to the patient's history of heart surgery, it was crucial to prioritize the exclusion of coughing symptoms caused by cardiac issues when assessing the cough. Coughing symptoms caused by heart failure may be related to posture (lying down) or exertion. They may also be accompanied by lower-limb edema. The side effects of blood pressure medications (ACE inhibitors) can also lead to chronic coughing. Therefore, when evaluating patients with chronic coughs, it is essential to first assess the patient's medical history and medication history. In this case, the onset of symptoms has been ruled out for cardiac, pulmonary, and respiratory-related pathological lesions by three TCM practitioners and a cardiologist.

Distinguishing between somatic chronic cough and non-somatic chronic cough can be challenging [5], as patients with chronic cough are more prone to experiencing psychological disorders such as anxiety and depression, which can, in turn, exacerbate the coughing. While it is commonly believed that somatic chronic cough tends to occur more frequently when patients are awake and less frequently during sleep, the presence of accompanying physiological conditions such as gastroesophageal reflux, postnasal drip, bronchitis, etc. can still result in pronounced nocturnal coughing [5]. The presence or absence of nocturnal cough cannot be used as the sole basis for diagnosis; somatic cough syndrome can only be diagnosed when a patient meets the Diagnostic and Statistical Manual of Mental Disorders, Fifth Edition (DSM-5) criteria [3,5]. In this case, although it is evident that the cough has persisted for a long time and has caused significant stress, with a notable impact on the patient's emotions, there are no records of psychiatric assessments before or after the onset of symptoms. Therefore, it is not possible to diagnose or rule out somatic chronic coughs solely caused by depressive emotions. Additionally, the nature of the cough, with the patient experiencing almost no symptoms during the day and only coughing when lying flat in bed during her deep sleep, does not align with typical somatic cough characteristics. Instead, it resembles conditions previously diagnosed by three TCM practitioners, such as postnasal drip and gastroesophageal reflux. Although these possibilities have been explored in treatment, there has been no significant improvement. Therefore, the cough is more likely to be diagnosed as a chronic neurogenic cough.

Oversensitivity of the vagus nerve is considered one of the main factors in chronic neurogenic cough [3,13]. The Arnold nerve reflex induces a cough reflex by stimulating a branch of the vagus nerve in the ear. This branch runs from the vagus nerve to the external auditory canal, making it the only area where the vagus nerve is superficially distributed [3,13]. Testing primarily involves inserting a cotton swab into the external auditory canal for 3-5 mm and gently rotating it for two to three seconds. If coughing occurs within 10 seconds, it is considered a positive response. Studies have found that in adults with chronic cough, the prevalence of the Arnold nerve reflex is over 11 times higher compared to healthy individuals, especially in females. However, the prevalence of this reflex in pediatric patients with chronic cough did not show a similar increase, suggesting that vagus nerve hypersensitivity may be acquired later in life [13]. The authors of this study suggest that the mechanisms of chronic cough in adults and children may differ. The hypersensitivity of the vagal response in adults might be related to acquired infections or other environmental factors later in their lives, although there is currently no supporting research confirming infections as the main cause. In light of these findings, further research is needed to investigate whether adult chronic cough, possibly induced by cervical dysfunction, which is more commonly seen in adults, could be linked to an overactive vagus nerve. A case report published in 2020 identified three patients with chronic neurogenic cough, in whom coughing was triggered by hair stimulation of the vagus nerve within the ear canal. Significant improvement in coughing was observed after the removal of the hair, indicating that the over-activation of the vagus nerve plays a significant role in unexplained chronic cough [14].

The improved chronic cough in this case may be related to adjusting the upper cervical spine to affect the balance of the autonomic nervous system and/or regulating emotional stress. The vagus ganglia consist of the superior ganglion, the jugular ganglia, and the inferior ganglion, the vagal nodose ganglion, which primarily innervates the trachea and lungs, responding to mechanical stimuli. Both ganglia are closely interconnected and situated between the occiput (C0) and the first cervical vertebra (C1). In guinea pig studies using selective stimulation of C-fiber subtypes of the vagus nerve, it was shown that activation originating from the vagal nodose ganglion can acutely inhibit coughing, while activation from the jugular ganglia makes coughing more sensitive or triggers coughing [15]. In other words, C fibers originating from different ganglia may play opposite roles in cough regulation, and it is possible that humans have similar opposing regulatory mechanisms [15]. The joint dysfunction between the occiput and the first cervical vertebra may disrupt the normal functioning of the nerve ganglia. In this case, the first adjustment focused on correcting C0/C1, and after the first adjustment, a noticeable improvement in nocturnal coughing and restored C0/C1 joint play were observed. It is hypothesized that this change in the relative position or restored joint play of the cervical vertebrae may have an impact on the vagus nerve.

Some studies have also found that relative head posture can impact vagal tone, thereby influencing respiratory and cardiac functions. The study suggests that lying flat increases vagal activity, potentially triggering bronchoconstriction mechanisms in asthma patients. Tilted at 60 degrees, vagal activity decreases, aligning with Mossberg's 1990 hypothesis that vagal nerve activation in the supine position may be associated with nocturnal respiratory symptoms in asthma patients [16]. Another study assessed the impact of head positioning on the proportion of atrial fibrillation episodes in patients with atrial fibrillation. The results showed that tilting the head down increased vagal tone, leading to a decrease in fibrillation rate, while tilting the head up increased sympathetic activity, resulting in a higher fibrillation rate [17]. In a study published by Moustafa et al. in 2020, participants with a forward head posture demonstrated abnormal sensory-motor control and dysfunction in the autonomic nervous system compared to those with a normal head alignment [18]. These indirect pieces of evidence imply that in cases of cervical dysfunction, changes in the relative position of the head and neck by the patient may interfere with the vagus nerve and impact the automatic nervous system. This may explain the absence of coughing symptoms when the patient is in an upright position during the day. However, at night, when lying down to sleep, alterations in the relative position of the head and neck impact her automatic nervous system, leading to intense coughing.

The vagus nerve, in addition to controlling the cough reflex, is also a crucial component of the autonomic nervous system and is closely associated with the body's response to emotional stress [3,8,19]. Apart from physical/mechanical stimuli, the vagus nerve can also be influenced by chemical/hormonal stimulation. Stress can stimulate the hypothalamus-pituitary-adrenal axis or act through the sympathetic-adrenal-medullary system, involving the sympathetic nervous system, adrenal glands, and medullary system, to produce stress hormones. Under prolonged stress, the body releases large amounts of adrenaline and other stress hormones, altering the immune system and making the body more susceptible to inflammation and heightened sensitivity to pain stimuli [20]. The exact mechanisms behind this process are not fully understood, but it appears to contribute to an increased sensitivity of the cough reflex. Over time, coughing may become a habit, even evolving into a physiological and psychologically conditioned reflex state. Therefore, resetting this mind-body response through cognitive-behavioral therapy can be an effective therapeutic approach [3,7]. Spinal adjustments have also been found to regulate the autonomic nervous system. In healthy subjects, receiving adjustments in the upper cervical spine (C1/2) activates the parasympathetic nervous system [21], while adjustments in the lower cervical spine (C6/7) activate the sympathetic nervous system. For patients with neck pain, adjustments in either the upper or lower cervical spine activate the parasympathetic nervous system [21]. Balancing the autonomic nervous system and regulating stress can be a viable direction for treating chronic cough.

Furthermore, chronic cough patients have a higher prevalence of symptoms related to autonomic nervous system dysfunction and depressive symptoms [4,22]. It remains unclear whether these are caused by coughing or if chronic coughing and depression are actually part of a broader pathological manifestation of vagus nerve dysfunction. In recent years, vagus nerve stimulation has been increasingly used to treat severe depression and has shown considerable effectiveness [23]. In addition to implanting electrode chips into the vagus nerve in the neck, research has found that electrical stimulation of the Arnold nerve, a branch of the vagus nerve in the ear, can improve depressive symptoms [24]. This is believed to be related to the vagus nerve's regulation of and reduction in the body's inflammatory response. In animal experiments with rats, continuous vagus nerve stimulation was found to increase the secretion of serotonin and norepinephrine in rats [25]. Serotonin has been found to have inhibitory effects on cough reflexes in the peripheral or central nervous system [6]. This is one of the mechanisms underlying the improvement in neurogenic chronic cough patients when given antidepressant medications. Once again, it suggests that vagal nerve dysfunction may be associated with chronic coughing and depressive symptoms to some extent.

People with self-harming thoughts may not necessarily disclose their true feelings to others, especially when chiropractors are not specifically trained to address such issues. The identification of these thoughts was actually made through the OD method. Subsequently, using OD to assess the appropriateness of addressing and adjusting these mind-body connections was undertaken. If OD indicates adjustability, the process continues; however, if OD shows an inability to adjust, the process will not continue, and an urgent referral to a psychiatrist is necessary (Figure 2). This is indeed a serious matter. With the patient's consent, communication was established with both the patient and her family, elucidating the severity of the situation and seeking family support in consulting with a mental health professional. Documenting the findings and reactions is crucial. Research has shown that individuals with a history of depression are more likely to experience a resurgence of depressive symptoms in the context of chronic cough [4]. This phenomenon was also observed in this case and significantly impacted her subsequent recovery. According to the biopsychosocial model, especially for individuals with chronic health issues, a multidisciplinary professional intervention is necessary [26]. Approaching the chronic situation solely from a physiological perspective often yields limited results. Some chiropractic techniques incorporate psychological aspects, but their primary objective remains to alleviate musculoskeletal discomfort. It is not intended as a direct treatment for the patient's mental health issues. When encountering such cases, it is essential to refer the patient for further assessment and treatment by mental health professionals.

Limitations and suggestions

The assessment of cervical X-rays or autonomic nervous system testing is not a routine practice for chronic cough patients or those with simple neck pain lacking red flags or neurological deficits. Due to the patient's preference to avoid non-essential medical examinations, there was no deliberate acquisition of objective measurements before treatment, such as cervical X-rays, autonomic nervous system testing, stress hormone levels, pulmonary function tests, etc., for a comparative pre- and post-analysis. Therefore, it is also impossible to determine whether the effect is solely from spinal manipulation, the KST emotions protocol, a combined effect of both, or just a placebo effect. While this case shows surprising improvement, relying solely on subjective improvement without objective evidence is still insufficient scientifically. For a more comprehensive understanding, further research could explore upper cervical dysfunction and its potential role in vagus dysfunction and stress hormone influence on chronic neurological cough based on this case. Until there is clearer evidence for the treatment of chronic cough, it cannot replace existing therapies. Chiropractors should continue to focus on treating musculoskeletal system imbalances.

## Conclusions

The treatment of chronic cough can be frustrating for patients, especially when many find conventional medications ineffective or experience bothersome side effects. This often results in significant psychological and social challenges. From a psychoneuroimmunological perspective, individuals with chronic coughs may experience stress at both neural and psychological levels. While chiropractic care primarily focuses on improving musculoskeletal issues and inducing relaxation, the additional improvement in chronic cough following adjustments to the upper cervical spine and KST emotions protocol provides a new perspective on the underlying mechanism. It is worth considering this as a starting point for further exploratory research.
